# Book Review

**Published:** 1938

**Authors:** 


### Reviews of Books

**The Doctrine of Signatures.** By S. Buchanan. Pp. xvi., 205. London: Kegan Paul, Trench, Trubner & Co. Ltd. 1938. Price 7s. 6d.—The author of this book is the Professor of Philosophy in the University of Virginia. He has turned aside for a short while to make a study of the study of medicine. He does not pretend to any knowledge of the practice of the medical art. He places medicine at the head of the Natural Sciences. "It has," he says, "a record maximum of knowledge and a minimum of wisdom, to say nothing of practical and philosophical wisdom." He offers this doctrine of signatures to satisfy "our vague yearnings for an integral medical science." It is not quite clear who has these vague yearnings; however, he puts forward the doctrine because "it says that medical knowledge and skill consists in seeing the connections between symptoms and remedies." He gives the illustration that it is "easier to see the signatures themselves . . . as when the extract of the heart-shaped leaves of the foxglove is called the specific for angina pectoris." This he terms "a classic illustration." It seems almost sacrilegious to peer any further into this mystic symbolism! Professor Buchanan's historical account of the development of the liberal arts is interesting, especially when he traces out the successive meanings that have attached to the word "grammar," and its relation to "rhetoric" and "logic," the other two elements of the *trivium*. The modern doctor is credited with a larger acquaintance with the methods of Aristotle and Galen than he can claim. Professor Buchanan tells us that according to Galen's teaching "the physiologist detects the structure of the hierarchy of forms in any organ as its four causes, material, formal, efficient and final." Galen may indeed have contributed something to the evolution of medicine but his contribution was certainly not contained in jargon of this sort. Professor Buchanan claims that "Aristotle and Galen are the mediums through which we see, often unawares, not only our scientific problems of the present, but even the problems of common sense that any practiser of the medical arts has to face daily." Somehow the thread of Greek and Hippocratic medicine seems to pass through the hands of the artists like Pheidias and Praxiteles to a Leonardo da Vinci rather than through the minds of philosophers. It is doubtful if Galen enriched medicine at all, even though he may have preserved some knowledge. After Leonardo came Vesalius and to him succeeded Harvey—these men laid the foundations of our modern medical art because they were artists, not because they were philosophers. Medicine owes little or nothing to the "systems" which men have invented for its study and practice. Philosophy and metaphysics have added nothing to our knowledge and understanding of medicine. Professor Buchanan might do worse than abandon his contemplation of "signatures" and study the effects on medicine of Auenbrugger's knowledge of music, and of Pasteur's accomplishments in the art of drawing and painting. Galton knew the value of this artistic sense to scientific progress. He found in youth a high proportion who exhibit the faculty of mental imagery and artistic perception. He deplored that this faculty which gives accuracy to our perceptions and justness to our generalizations runs a risk of being suppressed by our bookish and wordy education and of being deliberately starved by the lazy disuse of philosophers. Is it unjust to Professor Buchanan to suggest that his *Doctrine of Signatures* is bookish and wordy?

**The History of the Forceps.** By V. Møller-Christensen. Pp. xx., 298. Illustrated. London: Oxford University Press. 1938. Price 20s.—This interesting historical study is translated from the Danish. There are upwards of 400 illustrations, and the writer has travelled all over the world to obtain his material, as well as making a special study of early literature. The first surgical forceps on record was produced in Egypt about 3300 b.c. This appears to be an original invention. There were forceps in use in Denmark in the Bronze Age. The evolution of the artery forceps is also given consideration.

**Text-book of Nutrition.** By J. A. Nixon, C.M.G., M.D., F.R.C.P., and D. G. C. Nixon, M.B., B.S., M.R.C.P., L.R.C.P. Pp. xiv., 219. London: Oxford University Press (Humphrey Milford). 1938. Price 7s. 6d.—The subject of nutrition must always be of first importance to all men and women, nations and, indeed, the whole animal kingdom. Yet there is probably no subject on which more rubbish has been written and said, not only by the advocates of various fads, but even by those

K

who ought to be better informed. We can therefore heartily welcome this exceedingly valuable account of the *known facts*. As the authors are careful to point out, nutrition embraces a great deal more than mere food and drink : but naturally these occupy the main bulk of the book. Here one can find in convenient form a short summary of the processes of digestion and absorption. In tabular form we can read off the exact composition of every ordinary food—calories, first-class proteins, vitamins, and minerals. Nor has the preparation of food been neglected : the changes due to cooking or other preparation are given, and, what is very important, the cost of various diets, including the cost of preparation. The authors point out how extremely diverse are the diets that are apparently compatible with perfect health : comparing not only race with race but different individuals of the same race and social standing. A short chapter is devoted to diet in disease ; if we have a suggestion to offer it is that a dietary suitable for those who can only swallow fluids would fill a long-felt want. The book is carefully printed and well indexed, and in an admirable manner fills a real need.

**Faith Healing.** By C. W. J. BRASHER, M.D. Pp. 204. London: Gerald Duckworth & Co. Ltd. 1938. Price 5s.—Dr. Brasher has collected from all over the world instances of spontaneous cures of disease in unexpected cases. On the wrapper the publishers say that the object of the book is to show that the teachings of modern psychology are in no way incompatible with the belief in "Faith Healing." It would be unnecessary to contest this opinion. There is considerable philosophical and metaphysical speculation in the book and a whole-hearted endeavour to show the importance of the psychological approach to persons in ill-health. No experienced practitioner, whether orthodox or unorthodox, has ever doubted this. A good many instances of spontaneous cure in unexpected cases are cited : the examples are interesting, the explanations are conjectural. In an appendix full reports are published of five "cures" which occurred at Lourdes. These reports are long and verbose ; there is no doubt that the patients declared themselves instantaneously cured. A careful study of the reports rouses considerable doubt whether the patients might not equally well have declared themselves cured before their visits to Lourdes had they been so minded. Since they were not so minded, the Lourdes visits were worth while. Dr. Brasher's collection of spontaneous cures in unexpected cases, which he regards as illustrations of "faith-healing," recalls Diday's plea for recording "those rare cases which are registered in the annals of science to render diagnosis more circumspect."

**Myocarditis** (The St. Cyres Memorial Lectures). By J. S. GOODALL, F.R.C.S., F.R.S., and others. Pp. 152. London: Eyre & Spottiswoode Ltd. 1937. Price 10s. 6d.—In 1926 the late Viscountess St. Cyres founded and endowed, in memory of her husband, the St. Cyres Lectures on the Subject of Myocarditis, to be given annually. Six of the first seven lectures have now been published in the present volume. Unfortunately, no record has been kept of the fifth lecture, given by Sir Thomas Lewis, and this has, therefore, had to be omitted. The lectures published include two by Dr. Strickland Goodall on "Some general aspects of Myocarditis" and on "the Right Side of the Heart." Dr. R. O. Moon also deals generally with Disease of the Myocardium, and Dr. John Cowan "the Fibroses of the Heart," while a valuable lecture by Professor John Hay describes "Certain Aspects of Coronary Thrombosis." Perhaps the most welcome of all to English-speaking readers will be Professor Wenckebach's contribution on the "Heart and Circulation in a tropical avitaminosis (Beri-beri)." All of these lectures will repay careful study and the volume will be a useful addition to the book shelves of all those interested in diseases of the heart.

**Modern Treatment in General Practice.** Vol. IV. Edited by C. P. G. WAKELEY, D.Sc., F.R.C.S., F.R.S.E., F.A.C.S., F.R.A.C.S. Pp. xii., 440. Illustrated. London: Baillière, Tindall & Cox. 1938. Price 10s. 6d.—These Modern Treatment Volumes—now to be made into "Annuals"—should make a strong appeal to the General Practitioner for whom they are designed. They ably span the gap between the stereotyped text books, which depend on the clarity of the style of a single author for success, and the specialized monographs. The 1938 volume is a sound, practical work, and in fifty-two chapters covers a wide variety of subjects in almost every branch of medicine and surgery, the information being provided in many instances by specialists of repute. Particular value attaches to the second part of the volume, "Pitfalls in Diagnosis," which provides information which can be put into daily use. This section deals with differential diagnosis, and as the first essential in treatment is accurate diagnosis, we would like to see the entire contents of future volumes written in this style. The book, as a whole, is well brought out and can be recommended with confidence to all medical men as a "refresher" and especially to the students of medicine and to the General Practitioners.

**The Evolution of Chronic Rheumatism.** By R. FORTESCUE FOX, M.D., F.R.C.P. Pp. 26. London: H. K. Lewis & Co. Ltd. 1938. Price 2s. 6d.—This expensive pamphlet is founded on a lecture delivered at the congress of the Royal Institute of Public Health a year ago. About ten pages are given up to discussing the causes of rheumatism, "rheumatic sequence," the simile of a spiral being used instead of the old vicious circle. There are useful diagrams of primordial causes. On the other ten pages the author airs his views on the treatment, and the necessity of observing the patient's reaction to changes of temperature is rightly stressed. A useful little pamphlet for spas and clinics.

**A Survey of Chronic Rheumatic Diseases.** Edited by R. G. GORDON, M.D., D.Sc., F.R.C.P., and others. Pp. viii., 338. Illustrated. London: Oxford University Press (Humphrey Milford). 1938. Price 18s.—The survey consists of a series of articles on the rheumatic diseases, contributed by contemporary authorities in commemoration of the bicentenary of the Royal National Hospital for Rheumatic Diseases, Bath. The group of physicians and pathologists in this country who are interested in the rheumatic diseases has been so active and clamant during the past two or three years that their views on this subject are generally well known, but although their contribution to this volume contains little that is new, it is an excellent summary of current opinion about these disorders. About half the content of the survey is contributed by medical authorities from America and the continent of Europe, and their varied views with regard to causation, pathology, and treatment will be read with the greatest interest. Hench's article, "Is rheumatoid arthritis a disease of microbic origin?" and Aschoff's on "Allergy in arthritis," are peculiarly thoughtful and suggestive. Whilst both are tentatively on the side of the microbial origin of rheumatic diseases, they reflect the general tendency of the present day to lay increasing emphasis on the importance of constitution, diathesis, and external factors. The infective theory seems fated ultimately to assume a secondary rôle. The contributions on the importance of biochemistry and radiology in the diagnosis of rheumatism, and those on the pharmacology and hydrology of chronic rheumatism, will be found to be of great interest and practical value.

**Introduction to Diseases of the Chest.** By J. MAXWELL, M.D., F.R.C.P. Pp. xii., 328. Illustrated. London: Hodder and Stoughton Ltd. 1938. Price 12s. 6d.—Dr. Maxwell designed this book for the student, and presents in it a study of the clinical aspects of respiration, the correlation of history with physical examination, and describes the various special examinations which are applicable to respiratory disease. He also devotes some attention to methods of treatment. The major symptoms are adequately set forth, but without giving any verbal picture of the patient such as Hippocrates, or in later days Sir Thomas Watson could so vividly depict. In the discussion of "physical examination," the principles of inspection are clearly enunciated, but the student is not advised as to which parts of the hand and fingers are best calculated to transmit information from palpation. Percussion seems to be dealt with in a wholly non-musical fashion; yet Auenbrugger based his discovery of the art of percussion on his musical knowledge. Is "resonance" solely a question of "pitch" and "duration" of a sound without reference to "loudness" or "intensity"? Is the physical state determined by percussion really the depth of the column of air which lies beneath the percussed part: if so, how are the kettledrums in the orchestra tuned to varying notes by merely altering the tension of the drum-head? Is "Skodaic resonance" proved to be due to relaxation of lung above a pleural effusion? Why is no reference made to the difference between the area of superficial cardiac dulness which demonstrates the extent of precordium uncovered by lung and the deep dulness which denotes the extent of heart underlying the lung? The chapter on auscultation bespeaks a want of acquaintance with Laennec's writings. A classification which says "the normal breath sounds are vesicular" is nonsense, nor is it true that "vesicular breath sounds are normally heard over the whole of the lungs with the exception of a small area in the second right space, close to the sternum." It is misleading to tell students that bronchial breathing is an abnormal breath sound. It is a normal sound which may be heard in abnormal places. The classification and description of added sounds are open to adverse criticism. Firstly because the meaning of the terms is not given, secondly because the explanation of why and where the several added sounds occur is by no means so certain as the author makes out, and thirdly because there is no justification for spelling *sibilus* "sibillus" as is done throughout the book. Is it fair to the student to tell him that succussion splash is pathognomonic of hydropneumothorax? Surely it does not exclude pus or a blood-stained effusion mixed with air. These may seem pedantic objections, but a teacher who is inexact in the use of words or ignorant of their meaning can hardly fail to mislead or confuse students who derive their instruction in clinical medicine from words spoken at the bedside or in the lecture-room. When one turns to the descriptions of special investigations, bacteriological, radiological and the like, the author becomes a more reliable guide. His directions for screening a chest and viewing a skiagram are workmanlike and practical. So are his occasional remarks on treatment, particularly his descriptions of lipiodol introduction and artificial pneumothorax. A good deal of attention is paid to the treatment of asthma, but no mention is made of the value of high altitude, especially in young asthmatics. Omitting the portions of the book which are open to criticism and appear limited to the first two sections, we find a great deal to praise in the accounts given of the various diseases and their treatment, and this includes five-sixths of the volume. Most of the faults lie in fifteen pages of Section 2, and there are three hundred and fifteen other pages which are very good.

**Sick Children.** Third Edition. By D. Paterson, M.D., F.R.C.P. Pp. iv., 604. Illustrated. London : Cassell & Co. Ltd. 1938. Price 12s. 6d.—That this book has reached a third edition within twelve months of the publication of the second edition is sufficient evidence of its general popularity. The excellent references and footnotes of original articles have been continued, whilst the appendix of recent examination papers will prove invaluable to students and those taking higher examinations in Children's Diseases. Advances in chemotherapy are very adequately dealt with, whilst the section on anaemia has been improved and a new footnote written on acute and chronic arthritis in infancy and childhood. Sonne dysentery has been particularly dealt with and special references made to epidemic jaundice. The illustrations and general production of this edition are very good indeed. The index is thoroughly adequate and the heavy type of the various headings makes reference easy. This book can be most heartily recommended to students and practitioners requiring concise and up-to-date information compiled in a most readable form.

**A Textbook of X-Ray Diagnosis by British Authors.** Vols. I and II. Edited by S. C. SHANKS, M.D., P. KERLEY, M.D., M.R.C.P., D.M.R.E., and E. W. TWINING, M.R.C.S., L.R.C.P., D.M.R.E. Pp. (I) xii., 591 ; (II) xii., 458. Illustrated. London : H. K. Lewis & Co. Ltd. 1938. Prices 50s. (Vol. I), 42s. (Vol. II).—This authoritative work is most comprehensive and supplies a much-needed want in British works on this subject. The numerous contributors have united to give the most reliable and up-to-date information on their respective subjects, in concise and lucid form. The preliminary notes given in each section on radiographic technique and the diagrams and notes on the anatomy of the part, are very clearly and concisely described and are exceedingly helpful. The illustrations are far superior to those found in most radiological text-books and are really excellent, great pains having obviously been taken to secure the best possible results. The most recent advances in radiographic technique are described and their practical application explained. Copious bibliographies are supplied. All the articles are written in a most readable style, and the excellent paper and print make them a pleasure to read.

**Anaesthesia and Analgesia for Nurses and Midwives.** By J. K. WATSON, M.D. Pp. viii., 135. Illustrated. Bristol : John Wright & Sons, Ltd. 1938. Price 3s. 6d.—This small book on anaesthesia by Dr. Watson is a concise and well-written elementary treatise on this subject. Although it is described as a manual for nurses and midwives, its pages contain quite sufficient information for the average medical student as well, and with an additional chapter on premedication, which is so much a part of anaesthetic routine nowadays, it should make a special appeal to him. The printing is clear and the illustrations are excellent, but A.C.E. as an anaesthetic mixture should surely find no place in a modern text-book.

